# Characterization and typology of beekeeping systems: a case study in the district of Tamburco, Apurímac, Peru

**DOI:** 10.3389/finsc.2026.1869943

**Published:** 2026-08-11

**Authors:** Alex Ñahui-Sayago, Delmer Zea Gonzales, Rutniss Alecy Vasquez-Onzueta, Nilton César Gómez Urviola, José Américo Saucedo-Uriarte, Lizeth A. Heredia-Vilchez

**Affiliations:** 1 Facultad de Medicina Veterinaria y Zootecnia, Universidad Nacional Micaela Bastidas de Apurímac, Abancay, Apurímac, Peru; 2 Facultad de Ingeniería Zootecnista, Biotecnología, Agronegocios y Ciencia de Datos, Universidad Nacional Toribio Rodríguez de Mendoza de Amazonas, Chachapoyas, Amazonas, Peru; 3 Grupo de Investigación en Biodiversidad, Taxonomía y Genética de Abejas (GIBITAGE), Instituto de Investigación en Ganadería y Biotecnología, Universidad Nacional Toribio Rodríguez de Mendoza de Amazonas, Chachapoyas, Amazonas, Peru; 4 Instituto de Investigación en Ganadería y Biotecnología, Universidad Nacional Toribio Rodríguez de Mendoza de Amazonas, Chachapoyas, Amazonas, Peru

**Keywords:** Apurímac, beekeeping, multivariate analysis, productivity, technification, typology

## Abstract

**Introduction:**

Beekeeping in rural regions of Peru presents considerable heterogeneity in technification and productivity, which may limit its development. This study aimed to characterize beekeeping systems and identify producer typologies associated with productive characteristics in the district of Tamburco, Apurímac, Peru.

**Methods:**

A quantitative, descriptive, cross-sectional study was conducted using a census sample of 34 active beekeepers. Data were collected through a structured and validated questionnaire. Data analysis included descriptive statistics, exploratory hierarchical cluster analysis, association tests, correlation analysis, and complementary multivariate analyses based on FAMD and HCPC.

**Results:**

The multivariate analyses identified distinct beekeeper typologies that differed in production scale, educational level, technification, and access to training. Significant associations were observed between cluster structure and several productive and sociodemographic variables, while beekeeping experience was positively associated with production scale.

**Discussion:**

Overall, the identified typologies were primarily associated with differences in production scale, technological level, and access to training. These findings may help guide extension and training strategies for beekeepers in Tamburco. Given the relatively small sample size and the cross-sectional design, the findings should be interpreted as exploratory and require validation in larger and geographically diverse populations.

## Graphical Abstract

**Figure:**
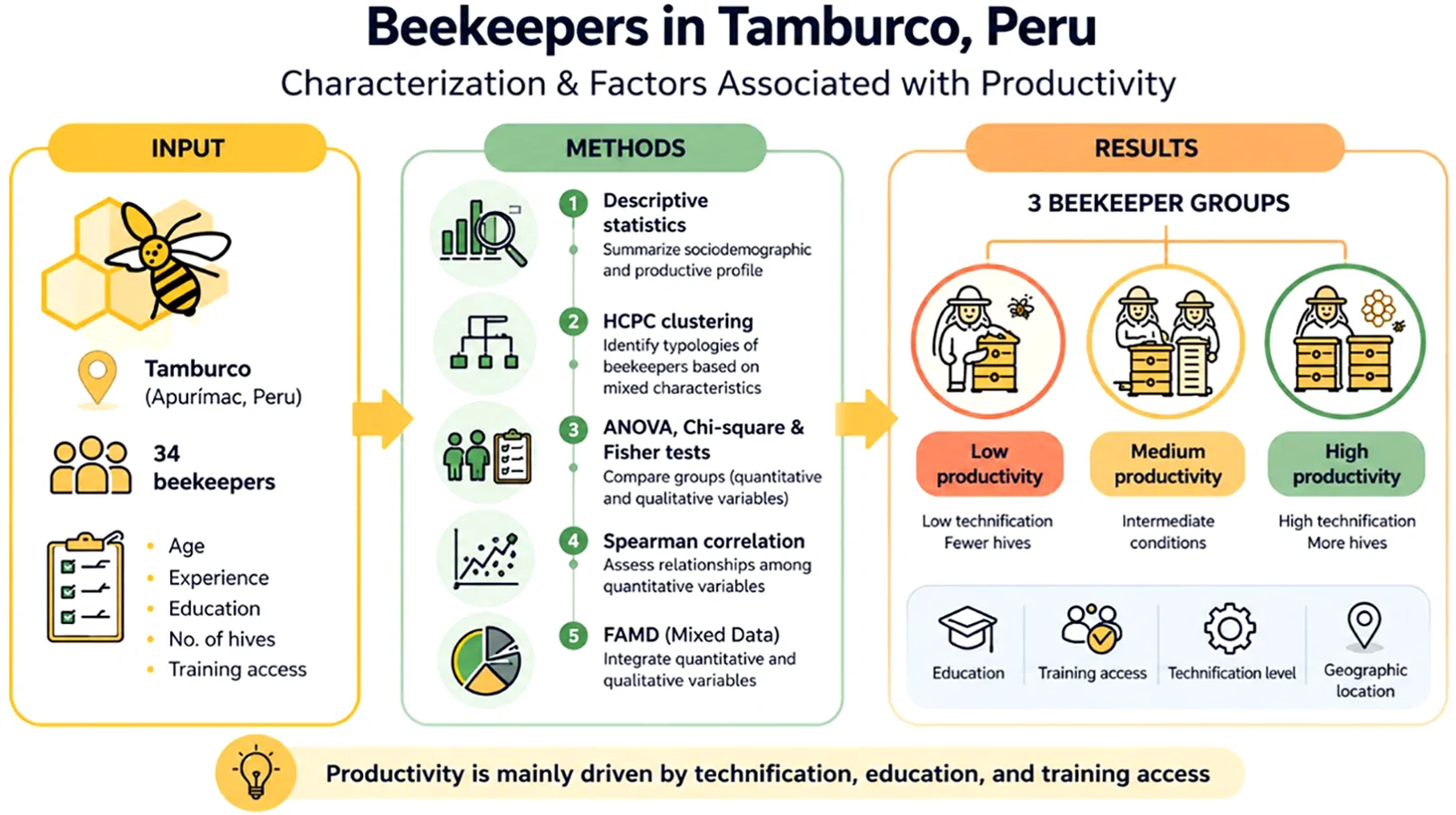

## Introduction

Beekeeping is a strategic activity for rural economies in Latin America, where it is predominantly carried out by small-scale producers managing fewer than ten hives (1). Despite its limited scale, this activity generates income and plays a key ecological role by contributing to pollination and ecosystem stability (2). In recent years, the beekeeping sector has experienced gradual development alongside increasing demand for honey and bee-derived products and the progressive incorporation of emerging technologies (2, 3).

Peru offers highly favorable conditions for beekeeping, with 84 life zones and more than 25,000 plant species that support rich apibotanical diversity (4). Peruvian honey is recognized for its nutritional and therapeutic properties, including antimicrobial, anti-inflammatory, and wound-healing effects (5). At the national level, the beekeeping sector has approximately 41,327 beekeepers, of whom nearly 95% belong to family farming systems (6), and 252,329 installed hives, of which 85% (214,276) are actively producing. The main producing regions are Cusco (11%), La Libertad (10%), Junín (9%), Lima (8%), and Apurímac (7%). The average production is 10.8 kg of honey per hive per year, reaching an estimated national production of 2,314 tons annually, with a projected productive potential of up to 500,000 hives (3, 7). However, despite this potential, important limitations persist in sanitary management, technification, and production practices, particularly among small-scale beekeepers (8, 9).

According to FAOSTAT (10), Peru ranked 74th worldwide in honey production in 2024, reflecting the relatively small scale of its beekeeping sector compared with the world’s leading honey-producing countries. Nevertheless, beekeeping plays an important socioeconomic role by supporting family farming and contributing to rural livelihoods in many regions of the country. In particular, the Apurímac region is not organized through formally established agricultural cooperatives; instead, the sector comprises approximately 15–25 beekeeper associations and local producer committees that operate in a decentralized manner.

Although formal agricultural cooperatives have not been established in the Apurímac beekeeping sector, producers are organized through local beekeeper associations and producer committees that facilitate technical support, training activities, and coordination among members. Therefore, the sector is characterized by a decentralized organizational structure rather than by formal cooperative schemes.

Honey production is influenced by a complex interaction of environmental, technical, and sociodemographic factors. Variables such as hive number, beekeeper experience, harvesting frequency, and level of dedication have been identified as key determinants of productivity. In addition, management practices such as nucleus preparation, brood frame management, queen replacement, and equipment disinfection are directly associated with colony health and honey yield (11, 12).

Among the main constraints in Peruvian beekeeping are diseases such as varroosis, American foulbrood, and nosemosis (13). In particular, infestation by *Varroa destructor* is considered a major global threat due to its negative effects on colony health and honey production (28, 14). Strong negative associations have been reported between infestation levels and productive performance, highlighting the importance of sanitary control measures to improve colony health and productivity. Additional limiting factors include pesticide exposure, *Nosema* infections, and reduced availability of floral resources (15).

Beekeeping systems are also highly sensitive to climatic variability, which affects bee physiology and floral resource availability, leading to fluctuations in production. Environmental factors such as temperature, humidity, solar radiation, and wind directly influence foraging activity and nectar availability. In addition, biodiversity loss caused by deforestation, agrochemical use, and vegetation burning further reduces melliferous flora and negatively impacts productivity (16, 17).

In Peru, state-funded beekeeping programs have been implemented; however, technical assistance has often been insufficient and poorly articulated with sector needs (18). This highlights the need to strengthen technical capacities, promote professionalization of beekeepers, and improve the adoption of good management practices to enhance productivity. In addition, territorial specificities should be considered in the design of interventions to ensure contextualized and effective strategies (11).

In the Apurímac region, beekeeping represents a potentially sustainable activity due to its ecological diversity and favorable environmental conditions. Nevertheless, the lack of systematized information on producer numbers, technification levels, floral availability, and productivity limits a comprehensive assessment of the sector (19). This gap restricts the design of evidence-based development strategies at regional and national levels (7).

Therefore, understanding beekeeper typologies is essential for identifying structural differences within production systems and for designing targeted interventions aimed at improving productivity and sustainability. In this context, the present study aimed to characterize and identify beekeeping typologies in the district of Tamburco, Apurímac, Peru, and to explore their association with productive characteristics in the sector.

## Methodology

### Study design and research area

**Figure 1:**
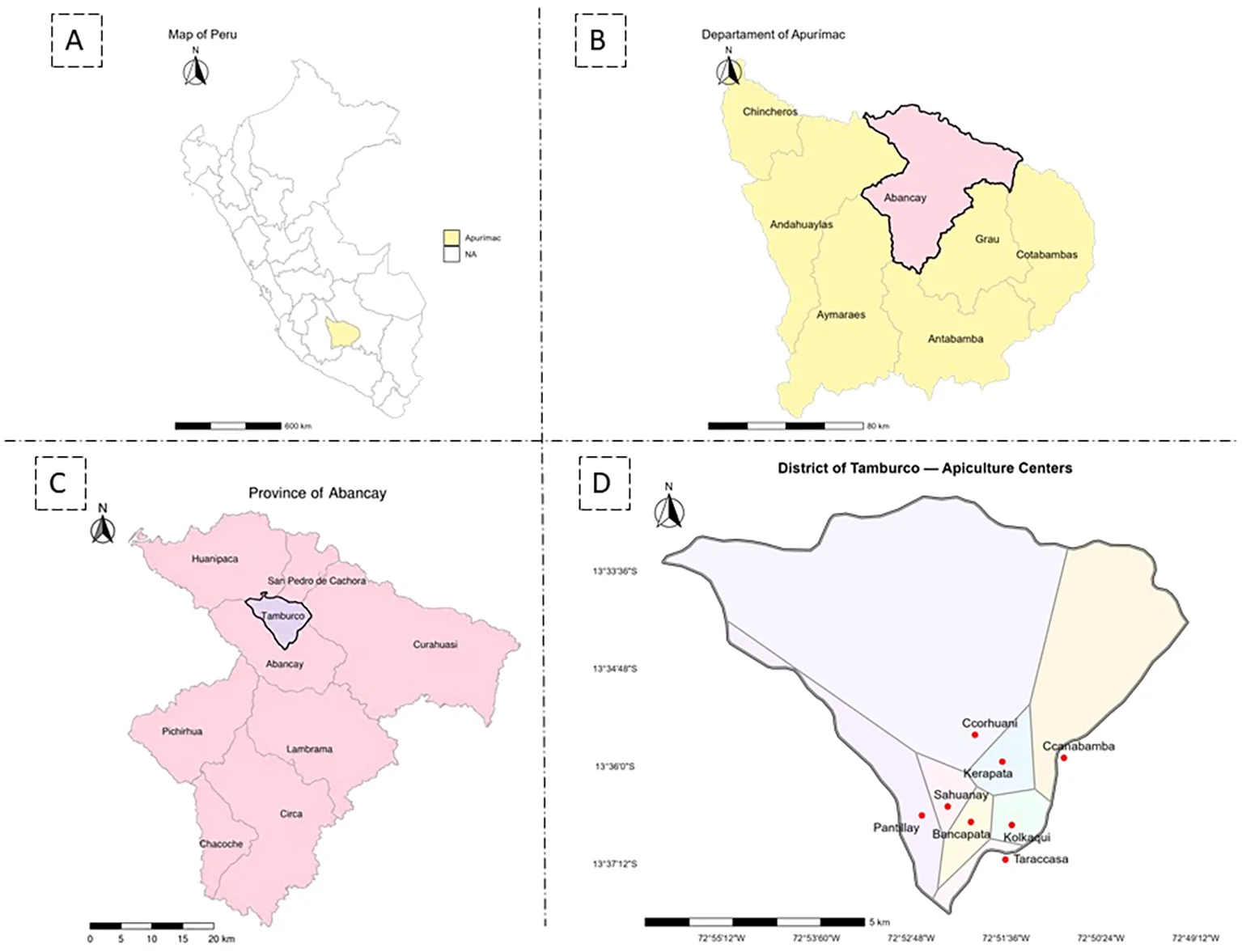
Geographic location of the study area and spatial distribution of surveyed apiculture centers in the district of Tamburco, Apurímac, Peru. **(A)** Map of Peru showing the location of the Department of Apurímac. **(B)** Map of the Department of Apurímac showing the location of the Province of Abancay. **(C)** Map of the Province of Abancay showing the location of the District of Tamburco. **(D)** Map of the District of Tamburco showing the location of the surveyed apiculture centers.

The study followed a quantitative approach, with a descriptive and non-experimental cross-sectional design. It was conducted in the district of Tamburco, province of Abancay, Apurímac region, characterized by a temperate climate and the presence of diverse melliferous flora, favorable for beekeeping activity. The area is located between 2,300 and 2,600 m above sea level, with average temperatures ranging from 15 to 22 °C and moderate relative humidity, conditions that favor foraging activity and the proper development of bee colonies. These environmental characteristics provide optimal conditions for hive management (**Figure 1**).

### Population and sampling

The study population consisted of all active beekeepers in the district of Tamburco during the study period. The official registry of the Regional Government’s “Proyecto Miel de Abeja” served as an initial reference to identify subsequently verified and complemented during fieldwork to ensure that all active beekeepers operating in the district were identified and included in the study. Consequently, a census sampling strategy was applied, resulting in a final sample of 34 beekeepers. Participation in the governmental project was not considered an inclusion criterion, and the study was not designed to evaluate the project itself.

### Data collection instrument

A structured questionnaire composed of 41 items was used, organized into five thematic sections: general beekeeper information, participation in projects, characteristics of beekeeping farms, hive management and health, and beekeeping production.

The instrument underwent a content validity process through expert judgment in order to evaluate the relevance, clarity, and coherence of the items. A pilot test was conducted to estimate the administration time, refine the wording, and ensure comprehension of the questionnaire.

The internal consistency of the instrument was assessed using Cronbach’s Alpha coefficient, which yielded a value of 0.992, indicating excellent reliability.

The complete questionnaire is presented as **Supplementary Material**.

### Data collection procedure

The surveys were conducted in person between February and April 2025. The collected data were recorded in a Microsoft Excel spreadsheet and subsequently cleaned for analysis.

### Data analysis

Data analysis was performed using R software (version 4.4.1; 20).

Initially, a descriptive analysis of the sociodemographic, productive, and management variables of the beekeepers was conducted using measures of central tendency and dispersion for quantitative variables and absolute and relative frequencies for qualitative variables.

As an exploratory step, a hierarchical cluster analysis based on the original variables was performed to obtain a preliminary characterization of the beekeeping systems and to identify general typological patterns among producers.

Based on the preliminary groups identified by the exploratory cluster analysis, quantitative variables were compared using one-way analysis of variance (ANOVA). Prior to ANOVA, the assumptions of normality of residuals and homogeneity of variances were evaluated. When significant differences were detected (p < 0.05), Duncan’s multiple range test was applied because the primary objective of this exploratory study was to identify homogeneous groups for biological interpretation. This procedure has been widely used in agricultural research for mean separation (21, 22). Qualitative variables were analyzed using Pearson’s chi-square test or Fisher’s exact test (p < 0.05), depending on compliance with statistical assumptions. Specifically, expected cell frequencies were evaluated, considering that no more than 20% of the expected frequencies were lower than 5 and none lower than 1. When these assumptions were not met, Fisher’s exact test was used. Independence of observations was ensured by the study design.

Relationships among quantitative variables were evaluated using Spearman’s rank correlation coefficient (p < 0.05).

Subsequently, a Factor Analysis of Mixed Data (FAMD) followed by Hierarchical Clustering on Principal Components (HCPC) was performed as a complementary multivariate analysis to further investigate the underlying structure of the data. Unlike the initial cluster analysis, which grouped individuals directly using the original variables, the FAMD transformed the same dataset into factorial components integrating both quantitative and qualitative variables, allowing a finer exploration of the multivariate heterogeneity among beekeepers.

Finally, the association between categorical variables and the HCPC clusters was evaluated using Pearson’s chi-square test or Fisher’s exact test, as appropriate, whereas the strength of association between quantitative variables and HCPC cluster membership was estimated using the eta-squared coefficient (η^2^).

## Results

### Sociodemographic and productive profile of beekeepers

The study population consisted of 34 beekeepers from the district of Tamburco. The mean age was 49.2 ± 13.4 years (range: 23–74 years), while the average beekeeping experience was 5.6 ± 7.3 years (range: 1–40 years). Most participants were male (82.4%), and the predominant educational level corresponded to basic education (41%). Regarding geographic distribution, the highest proportion of beekeepers was concentrated in Sahuanay (35%; n = 12), followed by Ccanabamba (18%; n = 6) and Pantillay (15%; n = 5), whereas the remaining localities showed lower representation (**Table 1**).

**Table 1:** Sociodemographic and productive characteristics of the beekeepers from the district of Tamburco.

Variable	Main result
Total number of beekeepers	34
Mean age	49.2 years (range: 23–74 years)
Mean experience	5.6 years (range: 1–40 years)
Sex	82.4% male (*n* = 28), 17.6% female (*n* = 6)
Educational level	41% basic education (*n* = 14), 29% secondary education (*n* = 10), 20% higher education (*n* = 7), 9% technical education (*n* = 3)
Geographic distribution	Sahuanay (*n* = 12), Ccanabamba (*n* = 6), Pantillay (*n* = 5), Ccorhuani (*n* = 4), Bancapata (*n* = 3), Kerapata (*n* = 2), Taraccasa (*n* = 1), and Kolkaqui (*n* = 1)

### Structural typology of beekeepers

**Figure 2:**
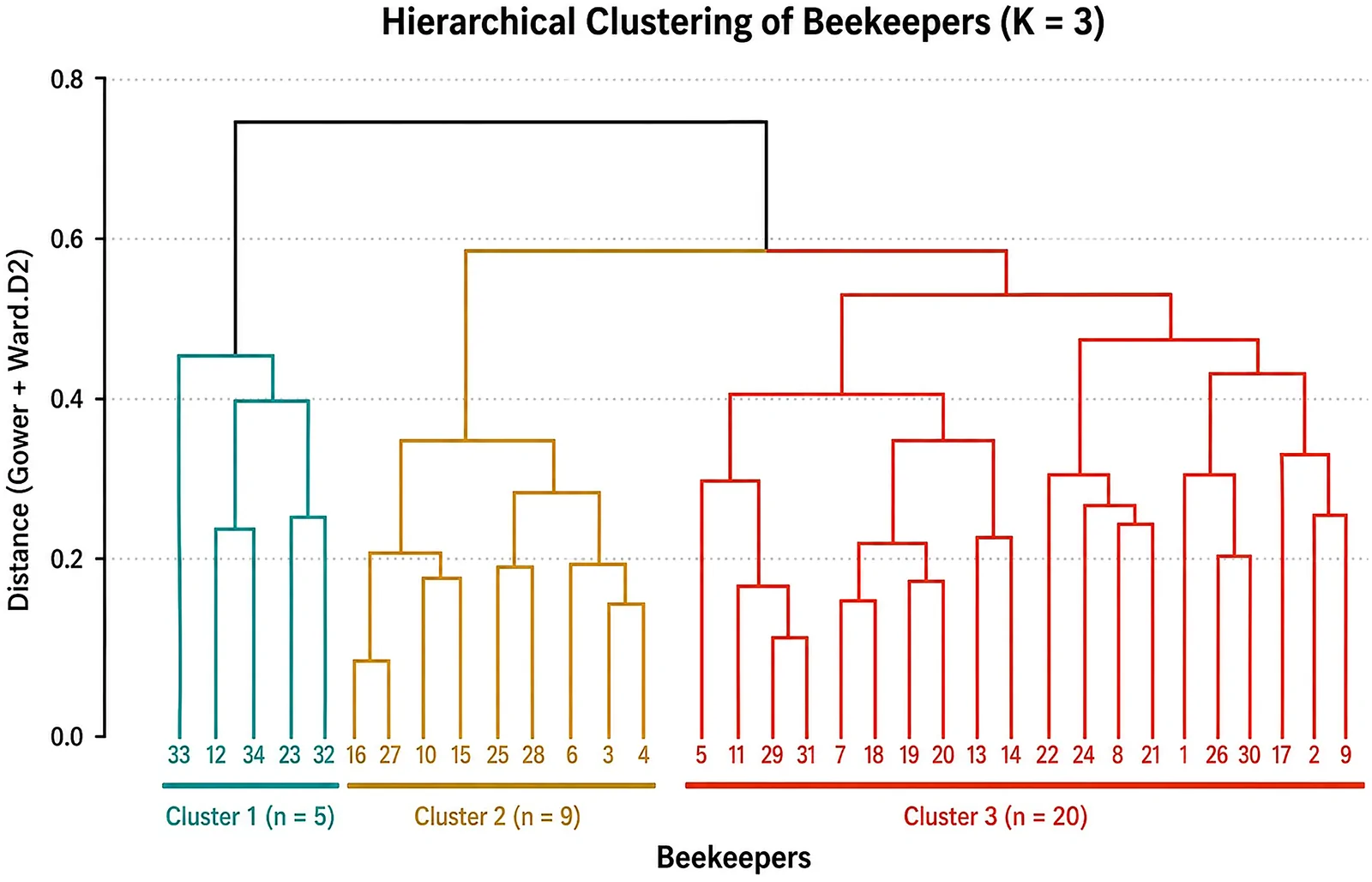
Hierarchical classification dendrogram of beekeepers in the district of Tamburco.

Hierarchical cluster analysis applied to the original variables provided an initial exploratory characterization of the beekeepers, identifying three preliminary groups with differentiated sociodemographic and productive characteristics (**Figure 2**).

### FAMD–HCPC multivariate analysis

To further investigate the multivariate structure of the data, a Factor Analysis of Mixed Data (FAMD) followed by Hierarchical Clustering on Principal Components (HCPC) was subsequently performed. Unlike the initial cluster analysis, which grouped individuals directly using the original variables, the FAMD transformed the same dataset into factorial components that simultaneously integrated quantitative and qualitative variables. The HCPC performed on this factorial space identified six statistically distinct clusters, revealing a finer level of heterogeneity among beekeepers than that observed in the initial exploratory analysis. This complementary approach provided a more detailed representation of the multivariate relationships within the study population. Euclidean distances used for cluster formation are presented in the **Supplementary Material** (**Supplementary Table 1**).

### Typological characterization of beekeepers

The main differential characteristics among the identified groups are presented in the **Supplementary Material** (**Supplementary Table 2**).

#### Typological comparison of beekeepers according to quantitative variables

When comparing productive variables among groups, only the number of supers (NA) and the number of hives (NC) showed significant differences (*p* < 0.01), whereas the remaining quantitative variables did not. In particular, the number of hives (NC) was significantly higher in Group 3, indicating that this group comprises beekeepers with the greatest productive capacity and the highest level of technological adoption. In contrast, variables such as age, experience, distance to the apiary, and the number of brood frames (CCP) did not vary significantly among groups (**Table 2**).

**Table 2:** Typological classification of beekeepers according to quantitative variables.

Variable	G1	G2	G3	F-value	p-value	Significance
Age (years)	51.2^a^	47.9^a^	43.2^a^	0.692	0.508	ns
Experience (years)	4.8^a^	4.0^a^	11.4^a^	1.551	0.228	ns
Distance to apiary (km)	4.12^a^	3.28^a^	2.50^a^	1.056	0.360	ns
Land area (ha)	1.45^a^	1.39^a^	1.40^a^	0.015	0.985	ns
Number of supers	1.20^a^	1.22^a^	2.00^b^	6.162	0.0056	*
Number of hives	6.00^a^	4.56^a^	76.00^b^	5.909	0.0067	*
Number of brood frames	2.00^a^	2.22^a^	2.00^a^	0.061	0.941	ns

Different superscript letters (a, b) indicate significant differences among groups according to Duncan’s multiple range test (p < 0.05). *ns,* not significant.

#### Typological comparison of beekeepers according to qualitative variables

Categorical variables were analyzed using Pearson’s chi-square test and Fisher’s exact test, as appropriate. Significant differences (*p* < 0.05) were found in variables related to educational level, the availability of floral resources, swarm type, and the level of census registration, indicating that the groups differ in both educational aspects and their beekeeping management strategies. In particular, groups with a higher educational level and a greater degree of formalization showed a stronger tendency toward more structured management practices, whereas traditional groups maintained a more empirical approach (**Table 3**).

**Table 3:** Typological classification of beekeepers according to qualitative variables.

Variable	G1	G2	G3	p-value	Sig.
Education	Predominantly basic	Predominantly basic	Predominantly higher	0.0129	Yes
Own capital	Yes	Yes	Yes	0.0049	Yes
Chacras (farmland plots)	High presence	Low	Low	0.0009	Yes
Chilca (*Baccharis latifolia*)	High	Low	Low	0.0051	Yes
Eucalyptus (*Eucalyptus globulus*)	High	High	High	0.0049	Yes
Avocado (*Persea americana*)	Low	Low	Medium	0.0372	Yes
Clover (*Trifolium* sp.)	High	Low	Medium	0.0170	Yes
Nabo (*Brassica rapa*)	High	Low	Low	0.0099	Yes
T**’**asta (*Escallonia myrtilloides*)	High	Low	Low	0.0058	Yes
Sauco (*Sambucus peruviana*)	High	Low	Low	0.0010	Yes
Borage (*Borago officinalis*)	High	Low	Low	0.0034	Yes
Sunchu (*Encelia canescens*)	High	Low	Low	0.0056	Yes
Swarm type: **“**Bóveda**”**	Low	Low	High	0.0205	Yes
Swarm type: **“**Cepa**”**	High	Low	High	0.0006	Yes
Swarm type: **“**Cucu**”**	Low	Low	High	0.0042	Yes
Swarm type: **“**Pal**”**	Low	Low	High	0.0052	Yes
Swarm type: **“**Jau**”**	Low	Low	High	0.0002	Yes
Swarm type: **“**Ext**”**	Low	Low	High	0.0012	Yes
Swarm type: **“**Ban**”**	Low	Low	High	0.0003	Yes
Census registration compliance	Low	Intermediate	High	0.0268	Yes
Formal beekeeping registration	Low	Low	High	0.0263	Yes

#### Correlation between sociodemographic and productive variables

**Figure 3:**
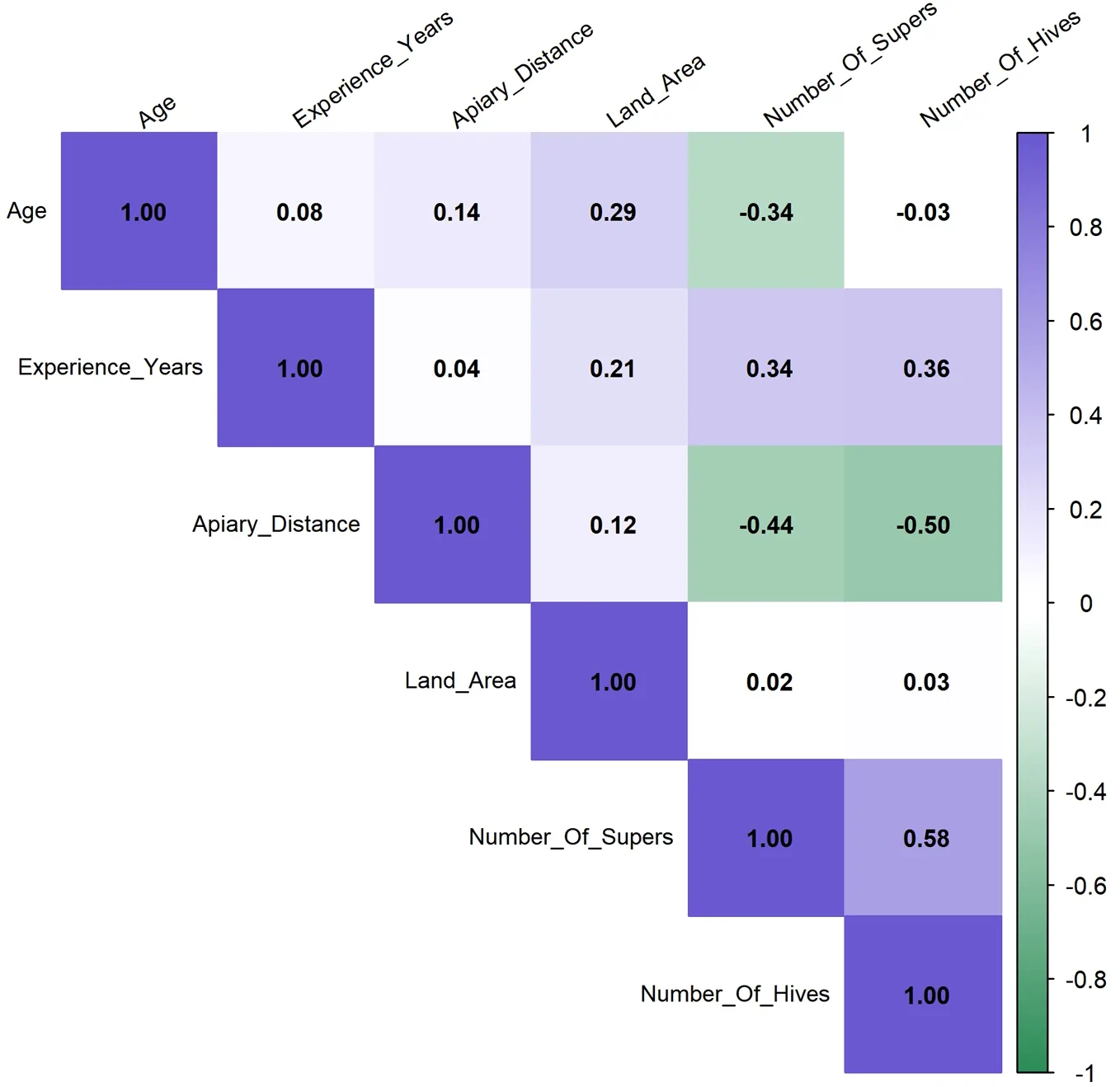
Spearman correlation heatmap showing the relationships among age, experience, apiary distance, land area, number of supers, and number of hives. Positive correlations are represented by blue tones and negative correlations by red tones.

Spearman’s correlation analysis revealed significant associations among some of the evaluated variables in beekeepers from the district of Tamburco (**Figure 3**; **Supplementary Table 3**). Positive correlations were observed between years of experience and number of supers (r = 0.34; p = 0.049), between years of experience and number of hives (r = 0.36; p = 0.039), and between number of supers and number of hives (r = 0.58; p < 0.001). Likewise, negative correlations were found between distance to the apiary and number of supers (r = −0.44; p = 0.009), and between distance to the apiary and number of hives (r = −0.50; p = 0.003).

The remaining correlations between variable pairs showed low coefficients and were not statistically significant (p > 0.05), including age with experience (r = 0.08), age with distance to the apiary (r = 0.14), age with land area (r = 0.29), age with number of supers (r = −0.34), age with number of hives (r = −0.03), experience with distance to the apiary (r = 0.04), experience with land area (r = 0.21), distance to the apiary with land area (r = 0.12), and land area with number of supers (r = 0.02) and number of hives (r = 0.03).

### Analysis using FAMD and HCPC

**Figure 4:**
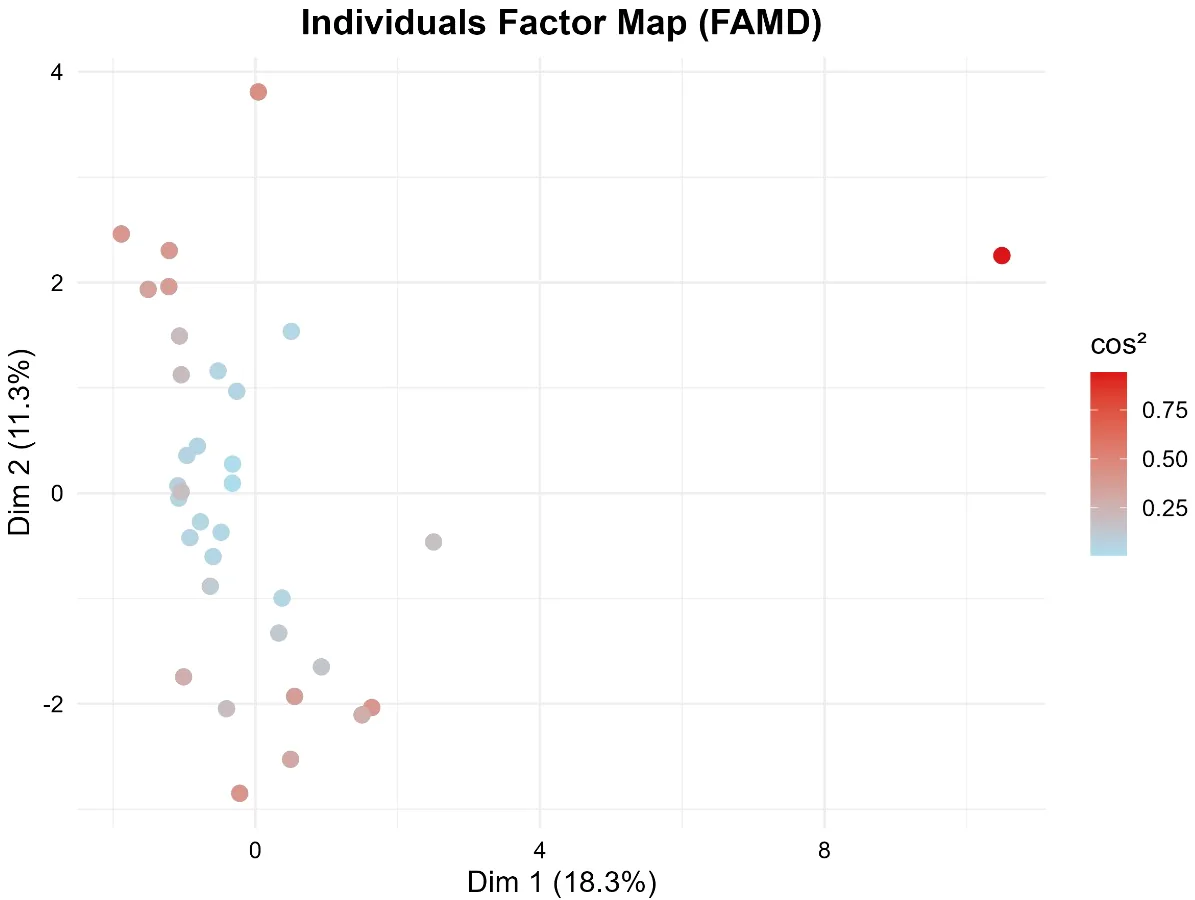
Individuals factor map based on FAMD. Each point represents an individual in the factorial space defined by the first two dimensions.

The FAMD analysis allowed the visualization of the distribution of beekeepers based on mixed variables (**Figure 4**). It was observed that most individuals are grouped around the origin, indicating relatively homogeneous profiles. However, one individual was clearly distinguished from the rest due to its particular characteristics. The variability observed in the main dimensions reflects the combined influence of both quantitative and qualitative variables.

**Figure 5:**
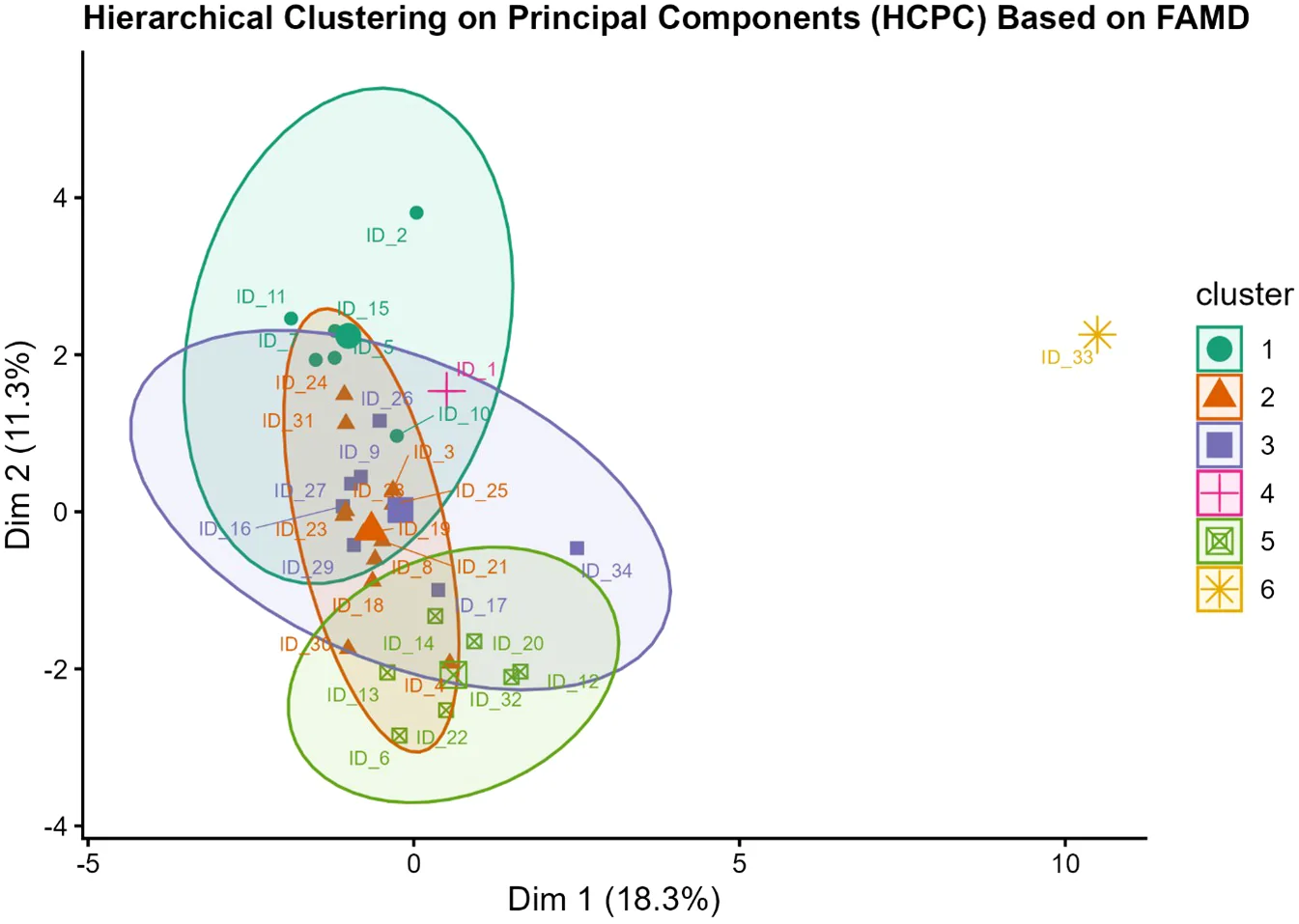
HCPC clustering based on FAMD. Points represent individuals and colors indicate clusters. Ellipses show group dispersion. Partial overlap among clusters is observed, with one clearly isolated individual.

The HCPC analysis based on FAMD identified six statistically distinct clusters in the factorial space, showing partial overlap among groups and a gradient-like structure rather than a strictly discrete partition (**Figure 5**). Although one individual appeared isolated in the factorial space, it was retained because it represented a valid observation within the study population and did not correspond to a data entry error.

Significant associations were found between clusters and categorical variables, including project benefits, geographic area, educational level, gender, and management stages (p < 0.05). In addition, quantitative variables showed strong associations with cluster structure, particularly number of hives (η^2^ = 0.94), age (η^2^ = 0.82), land size (η^2^ = 0.73), and number of supers (η^2^ = 0.46). A detailed description of cluster characteristics is presented in **Supplementary Table 4**.

### Association between variables

#### Association between categorical variables and cluster structure

Significant associations were observed between clusters and the variables project benefits, geographic area, education level, gender, and management stages (p < 0.05). No significant association was found between geographic area and ecological management (Fisher’s exact test, p = 0.7372). Likewise, no significant association was detected between the presence of varroosis and the use of fluvalinate (**Table 4**).

**Table 4:** Association between categorical variables and clusters (Chi-square and Fisher tests)^1^.

Variable/association	Test	Statistic	p-value
Project benefits vs clusters	χ^2^	15	3.69 × 10^–7^
Geographic area vs clusters	χ^2^	35	1.24 × 10^–6^
Education vs clusters	χ^2^	15	2.61 × 10^–5^
Sex vs clusters	χ^2^	5	3.12 × 10^–3^
Management stages vs clusters	χ^2^	5	2.34 × 10^–2^
Geographic area vs ecological management	Fisher	—	0.7372
Varroosis vs fluvalinate	Fisher	—	1.000

^1^χ^2^ = Chi-square statistic. The Fisher’s exact test does not report a test statistic. Statistical significance was considered at p < 0.05.

#### Correlation between quantitative variables

The correlation between beekeeping experience and the number of hives was evaluated (**Table 5**). A correlation coefficient of 0.355 was obtained with a p-value of 0.039.

**Table 5:** Correlation between quantitative variables (Spearman’s correlation)¹.

Variables	R	p-value
Experience vs number of hives	0.355	0.039

^1^ r = Spearman correlation coefficient. Statistical significance was considered at p < 0.05.

#### Relationship between quantitative variables and clusters (HCPC)

The relationship between quantitative variables and cluster structure was assessed using the Eta-squared coefficient (η^2^). Significant associations were observed for Hives (η^2^ = 0.9415), Age (η^2^ = 0.8233), Land (η^2^ = 0.7290), and Supers (η^2^ = 0.4603), all showing statistical significance (p < 0.05) (**Table 6**).

**Table 6:** Association between quantitative variables and clusters (HCPC, η^2^)^1^.

Variable	η^2^	p-value
Hives	0.9415	< 0.001
Age	0.8233	< 0.001
Land	0.7290	< 0.001
Supers	0.4603	0.0028

^1^ η^2^ represents the effect size and indicates the proportion of variance associated with cluster membership. Higher η^2^ values correspond to stronger associations between the quantitative variable and cluster membership. Statistical significance was considered at p < 0.05.

## Discussion

Cluster analysis allowed the identification of three groups of beekeepers with differentiated sociodemographic, productive, and technological characteristics. This structure suggests the presence of internal heterogeneity of the beekeeping system, in agreement with studies reporting that apiculture is not a homogeneous system but rather presents typologies associated with production scale and the level of technological adoption by producers (23, 24).

In the sociodemographic analysis, groups with lower levels of technification were composed of older producers with relatively less experience, whereas the group with higher productive development included younger producers with greater beekeeping experience. This pattern suggests generational differences in the organization of the production system and in the consolidation of the activity. Previous studies have reported that age and experience are associated with innovation adoption and the capacity to modernize beekeeping management practices (23).

Educational level also showed differences among groups, with a higher presence of higher education in the most technified cluster. This result is consistent with evidence indicating that education facilitates the adoption of technical, sanitary, and productive management practices by improving the ability to understand and implement innovations in rural systems (25).

Likewise, participation in training programs was concentrated in the most technified group, suggesting a relationship between access to technical extension services and the level of productive development. It has been noted that agricultural extension services and training programs can contribute to technology adoption and productivity by strengthening producers’ management capacities (26, 27).

The Chi-square association analysis showed significant relationships between cluster membership and variables such as project benefits, geographic area, educational level, gender, and management stages. These findings suggest that differences among beekeeper groups were associated not only with productive characteristics but also with social and organizational components of the beekeeping system, consistent with previous multivariate characterization studies in agricultural production systems (24).

In contrast, Fisher’s exact tests did not show an association between geographic area and ecological management (p = 0.7372), nor between varroosis and fluvalinate use (p = 1.000), indicating statistical independence between these variables under the analyzed conditions. Under the conditions evaluated in this study, no statistical association was detected between these variables. Therefore, management practices cannot be attributed to geographic location based on the present data alone but rather by individual producer decisions or their level of technical knowledge.

In the analysis of quantitative variables, Spearman’s correlation showed a moderate positive relationship between experience and number of hives (r = 0.355; p = 0.039). This positive association suggests that producers with greater beekeeping experience tended to manage a larger number of hives a greater number of hives, suggesting a progressive expansion process associated with the accumulation of practical knowledge in beekeeping activity (11).

Finally, the η^2^ analysis revealed a strong association between cluster structure and variables such as number of hives (η^2^ = 0.9415), age (η^2^ = 0.8233), land availability (η^2^ = 0.7290), and use of supers (η^2^ = 0.4603), all of which were statistically significant (p < 0.01). These results suggest that group differentiation was primarily associated with structural and productive variables by structural and productive factors of the system, particularly those related to production scale and resource availability.

The results suggest a heterogeneous structure of the beekeeping system, in which the identified typologies were primarily associated with productive, educational, and training-related variables, whereas territorial and specific management variables showed weaker statistical associations under the conditions evaluated. This behavior is consistent with studies indicating that beekeeping system organization is strongly associated with production scale, commercialization and the level of technological adoption by producers (23, 24).

Within the study population, the observed heterogeneity suggests that differences in human capital and access to technical support were associated with the identified typologies. However, these associations should be interpreted as exploratory and should not be generalized to other Andean beekeeping systems without additional evidence. Education, experience, and access to training may contribute to differences in management practices among the surveyed beekeepers. These findings may provide useful information for designing extension and training strategies in Tamburco, although additional studies are needed before extrapolating these recommendations to other regions. Accordingly, policy interventions in regions such as Apurímac should prioritize knowledge transfer, technical assistance, and organizational strengthening as central elements for enhancing the sustainability of the beekeeping sector.

Beyond technical and organizational factors, the availability and diversity of melliferous flora may also contribute to the expansion and sustainability of beekeeping systems by providing continuous floral resources for honey bees. Therefore, the conservation and restoration of native melliferous vegetation could represent complementary strategies to support small-scale beekeeping while promoting biodiversity conservation.

Although most surveyed beekeepers operate on a small scale, family-based beekeeping plays an important role in supporting rural livelihoods by providing complementary household income while contributing to crop pollination and biodiversity conservation. Furthermore, strengthening technical assistance, apiary registration, and producer organization may improve access to training, markets, and development opportunities for small-scale beekeepers.

## Limitations and perspectives

Our study has limitations that should be acknowledged. The analysis was restricted to a single district with a small sample size (n = 34), which may limit cluster stability, reduce statistical power, and make results sensitive to individual observations; thus, the proposed typologies should be considered exploratory. In addition, the cross-sectional design only allows correlational interpretations, not causal inference. Furthermore, no formal cluster validation or sensitivity analyses were performed, so the robustness of the HCPC structure requires confirmation in future studies. Finally, although sampling bias was minimized, external validation using larger and more diverse populations is necessary to confirm the generalizability and stability of the findings.

Future research should expand to larger and geographically diverse populations to improve the robustness and generalizability of typologies. Longitudinal designs are recommended to assess temporal dynamics and causal relationships.

## Conclusions

Three distinct clusters of beekeepers were identified, characterized by differences in production scale, beekeeping experience, educational level, and access to training. Clusters 1 and 2 were characterized by a small production scale (4–6 hives), limited experience, basic educational attainment, and the absence of formal training, whereas Cluster 3 exhibited a higher level of technification and a substantially larger production scale (76 hives).

Overall, the identified typologies were primarily associated with differences in production scale, technological level, and access to capacity-building programs, highlighting the potential relevance of extension services for the development of beekeeping systems. These associations should be interpreted as exploratory rather than causal, given the cross-sectional nature of the study. Furthermore, the relatively small sample size (n = 34) may limit cluster stability, external validity, and the generalizability of the findings beyond the study population.

Within the study population, the identified typologies provide an exploratory framework that may assist in designing extension and training strategies for beekeepers in Tamburco. Future studies including larger and geographically diverse populations are needed to validate these typologies before considering their broader application.

## Data Availability

The raw data supporting the conclusions of this article will be made available by the authors, without undue reservation.
