# Supplementary material for: Characterization and typology of beekeeping systems: a case study in the district of Tamburco, Apurímac, Peru
Source: Front Insect Sci. 2026 Aug 11;6:1869943. doi: 10.3389/finsc.2026.1869943 (PMC13504388; doi:10.3389/finsc.2026.1869943)
Supplement: Supplementary file 1 [file Table1.docx]

Table S1. Complete matrix of socioeconomic, productive, and beekeeping management variables used to calculate distances between producer groups.

| Full variable name | Code | Group 1 | Group 2 | Group 3 |
| --- | --- | --- | --- | --- |
| Area / Sector | Area / Sector | Sahuanay | Sahuanay | Sahuanay |
| Gender | Gender | Male | Male | Male |
| Age (years) | Age (years) | 51.25 | 47.89 | 43.20 |
| Beekeeping experience (years) | Beekeeping experience (years) | 4.80 | 4.00 | 11.40 |
| Educational level | Educational level | Primary / Basic | Primary / Basic | Higher education |
| Start of beekeeping activity | Start of beekeeping activity | Own initiative | Own initiative | Own initiative |
| Main occupation | Main occupation | Agriculture | Agriculture | Agriculture |
| Project beneficiary | Project beneficiary | No | No | Yes |
| Institution related to project | Institution related to the project | None | None | Regional government |
| Project benefits | Project benefits | None | None | Training |
| Beekeeper goals | Beekeeper goals | Increase the number of hives | Increase the number of hives | Increase the number of hives |
| Knowledge of management stages | Knowledge of management stages | No | No | Yes |
| Distance to apiary (km) | Distance to apiary (km) | 4.13 | 3.28 | 2.50 |
| Land area (ha) | Land area (ha) | 1.45 | 1.39 | 1.40 |
| Access to apiary | Access to apiary | Unpaved road | Unpaved road | Unpaved road |
| Land tenure | Land tenure | Own property | Own property | Own property |
| Water source: river | Water source: river | No | No | No |
| Water source: spring | Water source: spring | No | No | No |
| Water source: well | Water source: well | No | No | No |
| Water source: public network | Water source: public network | Yes | Yes | Yes |
| Water source: lagoon | FA_lag | No | No | No |
| Water source: irrigation canal | FA_can | No | No | No |
| Capital source | CP | Own capital | Own capital | Own capital |
| Melliferous flora: Chachacomo *(Escalonia resinosa)* | FM_cha | Yes | No | No |
| Melliferous flora: Chilca (*Baccharis latifolia*) | FM_chil | Yes | No | No |
| Melliferous flora: Eucalyptus (*Eucalyptus globulus*) | FM_euc | Yes | Yes | Yes |
| Melliferous flora: Molle (*Schinus molle*) | FM_mol | No | No | No |
| Melliferous flora: Avocado (*Persea americana*) | FM_pal | No | No | Yes |
| Melliferous flora: Clover (*Trifolium* sp.) | FM_tré | Yes | No | Yes |
| Melliferous flora: Turnip (*Brassica rapa*) | FM_nab | Yes | No | No |
| Melliferous flora: T’asta (*Escallonia myrtilloides*) | FM_tas | Yes | No | No |
| Melliferous flora: Sauco (*Sambucus peruviana*) | FM_sau | Yes | No | No |
| Melliferous flora: Borage (*Borago officinalis*) | FM_bor | Yes | No | No |
| Melliferous flora: Guava tree (*Psidium guajava)* | FM_gua | No | No | No |
| Melliferous flora: Plum (*Prunus domestica*) | FM_cir | No | No | No |
| Melliferous flora: Peach (*Prunus persica*) | FM_dur | Yes | No | Yes |
| Melliferous flora: Sunchu *(Encelia canescens)* | FM_sun | Yes | No | No |
| Apicultural flora: Alfalfa (*Medicago sativa*) | FA_alf | Yes | Yes | Yes |
| Apicultural flora: Huarango *(Prosopis pallida)* | FA_hua | No | No | No |
| Apicultural flora: Chincho (*Tagetes elliptica*) | FA_chi | No | No | No |
| Type of beekeeping | TC | Conventional beekeeping | Conventional beekeeping | Conventional beekeeping |
| Number of apiaries | NA | 1.20 | 1.22 | 2.00 |
| Number of hives | NC | 6.00 | 4.56 | 76.00 |
| Transhumance | RT | No | No | No |
| Hive type | TCA | American standard / Langstroth | American standard / Langstroth | American standard / Langstroth |
| Technical installation criteria | CT | Yes | Yes | Yes |
| Learning method | CIA | Self-taught | Training courses | Training courses |
| Bee race: Carniolan | RA_car | No | No | No |
| Bee race: Italian | RA_ita | No | No | No |
| Bee race: Creole | RA_cri | Yes | Yes | Yes |
| Bee acquisition | OA | Purchase of nuclei | Purchase of nuclei | Purchase of nuclei |
| Equipment: coverall | EI_mam | Yes | Yes | Yes |
| Equipment: veil | EI_vel | Yes | Yes | Yes |
| Equipment: boots | EI_bo | Yes | No | Yes |
| Equipment: gloves | EI_guan | Yes | Yes | Yes |
| Equipment: brush | EI_cep | Yes | No | Yes |
| Equipment: uncapping knife | EI_cuc | No | No | Yes |
| Equipment: hive tool | EI_pal | No | No | Yes |
| Equipment: smoker | EI_ahu | Yes | Yes | Yes |
| Equipment: queen cage | EI_jau | No | No | Yes |
| Equipment: honey extractor | EI_ext | No | No | Yes |
| Equipment: bench / support* | EI_ban | No | No | Yes |
| Equipment: frame lifter | EI_lev | Yes | No | Yes |
| Equipment: wax foundation | EI_cera | Yes | No | Yes |
| Inspection frequency | FR | Biweekly | Biweekly | Biweekly |
| Record keeping | CR | No | No | Yes |
| Presence of sick bees | AE | Yes | Yes | Yes |
| Varroosis | EC_var | Yes | Yes | Yes |
| Nosemosis | EC_nes | No | No | No |
| Foulbrood | EC_loq | No | No | No |
| Conventional control method | MC_con | Yes | Yes | Yes |
| Ecological control method | MC_eco | Yes | No | Yes |
| Fluvalinate | PU_flu | No | No | No |
| Amitraz | PU_ami | No | No | No |
| Thymol | PU_tim | No | No | No |
| Formic acid | PU_fór | No | No | No |
| Oxalic acid | PU_oxá | No | No | No |
| Tobacco | PU_tab | No | No | No |
| Botanical product | PU_pod | No | No | No |
| Syrup | PU_jar | Yes | Yes | Yes |
| Pest control method | PU_pla | Ash | Cleaning around the hive | Hive stands |
| Months of varroa presence | PU_mvar | December–March | December–March | December–March |
| Months of nosemosis presence | PU_mnes | No presence | No presence | No presence |
| Months of pest presence | PU_mpla | December–March | December–March | December–March |
| Lost hives | CCP | 2.00 | 2.22 | 2.00 |
| Honey production | PO_miel | Yes | Yes | Yes |
| Propolis production | PO_pro | No | No | No |
| Pollen production | PO_pol | No | No | No |
| Wax production | PO_cer | No | No | No |
| Swarm production | PO_enj | No | No | Yes |
| Queen production | PO_rei | No | No | Yes |
| Nucleus production | PO_núc | No | No | Yes |
| Hive rental | PO_alq | No | No | No |
| Commercialization: self-consumption | RC_aut | Yes | Yes | Yes |
| Commercialization: home sales | RC_cas | Yes | No | Yes |
| Commercialization: local market | RC_mlo | No | No | Yes |
| Commercialization: regional market | RC_mre | No | No | No |
| Commercialization: national market | RC_mna | No | No | No |
| Limiting factor | FLC | Rainfall | Diseases | Apicultural flora |
| Perceived profitability | AAR | Yes | Yes | Yes |

Table S2. Complete characterization of beekeeper groups from the district of Tamburco based on sociodemographic, productive, and management variables.

| Variable | Group 1 | Group 2 | Group 3 |
| --- | --- | --- | --- |
| Sociodemographic variables |  |  |  |
| Area | Sahuanay | Sahuanay | Sahuanay |
| Gender | Male | Male | Male |
| Age (years) | 51.25 | 47.89 | 43.2 |
| Experience (years) | 4.8 | 4 | 11.4 |
| Educational level | Primary | Primary | Higher education |
| Start in beekeeping | Own initiative | Own initiative | Own initiative |
| Main activity | Agriculture | Agriculture | Agriculture |
| Institutional participation |  |  |  |
| Project beneficiary | No | No | Yes |
| Institution | None | None | Regional government |
| Benefits | None | None | Training |
| Management stages | No | No | Yes |
| Productive characteristics |  |  |  |
| No. of hives (NC) | 6 | 4.56 | 76 |
| No. of supers (NA) | 1.2 | 1.22 | 2 |
| Brood frames (CCP) | 2 | 2.22 | 2 |
| Apiary distance (km) | 4.12 | 3.28 | 2.5 |
| Hectares | 1.45 | 1.39 | 1.4 |
| Hive type | Langstroth | Langstroth | Langstroth |
| Access to resources |  |  |  |
| Access | Unpaved road | Unpaved road | Unpaved road |
| Land tenure | Own | Own | Own |
| Water supply network | Yes | Yes | Yes |
| Predominant melliferous flora |  |  |  |
| Eucalyptus (*Eucalyptus globulus)* | Yes | Yes | Yes |
| Clover (*Trifolium sp.)* | Yes | No | Yes |
| Sauco *(Sambucus peruviana)* | Yes | No | No |
| Avocado *(Persea americana)* | No | No | Yes |
| Peach *(Prunus persica)* | Yes | No | Yes |
| Bee health |  |  |  |
| Varroa presence | Yes | Yes | Yes |
| Nosema | No | No | No |
| Foulbrood | No | No | No |
| Sanitary control | Yes | Yes | Yes |
| Production and diversification |  |  |  |
| Honey production | Yes | Yes | Yes |
| Pollen production | No | No | No |
| Wax production | No | No | No |
| Nucleus production | No | No | Yes |
| Queen production | No | No | Yes |
| Limiting factors |  |  |  |
| Main limiting factor | Rainfall | Diseases | Apicultural flora |

Table S3. Spearman correlation matrix among productive and sociodemographic variables

| Variables | Age | Experience | Distance | Land area | Number of supers | Number of hives |
| --- | --- | --- | --- | --- | --- | --- |
| Age | – | 0.08 | 0.14 | 0.29 | -0.34 | -0.03 |
| Experience | 0.08 | – | 0.04 | 0.21 | 0.34* | 0.36* |
| Distance | 0.14 | 0.04 | – | 0.12 | -0.44** | -0.50** |
| Land area | 0.29 | 0.21 | 0.12 | – | 0.02 | 0.03 |
| Number of supers | -0.34 | 0.34* | -0.44** | 0.02 | – | 0.58*** |
| Number of hives | -0.03 | 0.36* | -0.50** | 0.03 | 0.58*** | – |

***Note:*** *Values correspond to Spearman’s rank correlation coefficients (r).* p < 0.05; ** p < 0.01; *** p < 0.001.*

Table S4.Descripción de los clusters basada en variables categóricas y cuantitativas derivadas del análisis HCPC.

A. Categorical variables

| Cluster | Variable | Category | % in cluster | p-value | v-test |
| --- | --- | --- | --- | --- | --- |
| 1 | Education | Basic/Primary | 83.3% | 0.034 | 2.12 |
| 2 | Education | Secondary | 66.7% | <0.001 | 3.31 |
| 2 | Project benefits | None | 91.7% | 0.009 | 2.59 |
| 3 | Sex | Female | 71.4% | <0.001 | 3.52 |
| 3 | Project benefits | Technical assistance | 57.1% | 0.003 | 2.92 |
| 3 | Geographical zone | Kerapata | 28.6% | 0.037 | 2.08 |
| 4 | Geographical zone | Taraccasa | 100% | 0.029 | 2.18 |
| 5 | Education | Higher | 71.4% | 0.001 | 3.19 |
| 5 | Management stages | Yes | 85.7% | 0.004 | 2.87 |
| 6 | Project benefits | Equipment and materials | 100% | 0.029 | 2.18 |
| 6 | Geographical zone | Kolkaqui | 100% | 0.029 | 2.18 |

B. Quantitative variables

| Cluster | Variable | Mean (cluster) | Mean (overall) | p-value | v-test |
| --- | --- | --- | --- | --- | --- |
| 1 | Age | 68.2 | 49.2 | <0.001 | 3.64 |
| 1 | Land size | 2.67 | 1.43 | <0.001 | 3.63 |
| 1 | Distance | 6.08 | 3.66 | 0.007 | 2.70 |
| 2 | Land size | 0.92 | 1.43 | 0.017 | -2.38 |
| 4 | Land size | 4.00 | 1.43 | 0.005 | 2.83 |
| 5 | Age | 29.1 | 49.2 | <0.001 | -4.22 |
| 6 | Number of hives | 280 | 15.9 | <0.001 | 5.56 |
| 6 | Number of supers | 3 | 1.32 | 0.001 | 3.18 |
| 6 | Experience | 27 | 5.56 | 0.008 | 2.64 |

*Note: Only statistically significant variables (p < 0.05) are shown. Positive v-test values indicate overrepresentation within clusters, while negative values indicate underrepresentation.*
